# Preclinical evidence and possible mechanisms of β-asarone for rats and mice with Alzheimer’s disease: A systematic review and meta-analysis

**DOI:** 10.3389/fphar.2022.956746

**Published:** 2022-08-31

**Authors:** Xin-Yuan Du, Yu-Shuang Cao, Juan Yang, Li-Chen Guo, Tong Zhang, Qing Yuan, Xi Chen, Li-min Hu

**Affiliations:** ^1^ Tianjin University of Traditional Chinese Medicine, Tianjin, China; ^2^ Pharmacology, State Key Laboratory of Component-based Chinese Medicine, Institute of Traditional Chinese Medicine, Tianjin University of Traditional Chinese Medicine, Tianjin, China; ^3^ School of Health, Brooks College (Sunnyvale), Milpitas, CA, United States; ^4^ Epidemiology and Statistics, School of Public Health, Medical College, Zhejiang University, Hangzhou, China

**Keywords:** β-asarone, Alzheimer’s disease, preclinical evidence, mechanisms, meta-analysis

## Abstract

**Background:** Currently, there are many different drugs to improve Alzheimer’s disease (AD) from different pathways. As a supplement and alternative medicine, traditional Chinese medicine (TCM) targets multiple pathways which may be different from classical Western medicine, which may be orchestrated with Western medicine to materialize multiplying efficacy in AD patients.

**Objective:** To investigate the therapeutic effect and assess the available preclinical evidence and possible mechanisms of β-asarone which was extracted from Acorus gramineus Soland (Araceae, AGS) for AD based on rat and mouse animal models.

**Methods:** PubMed, Embase, Scopus, Cochrane Library, BIOSIS Previews, Web of Science, EBSCO, and Google Scholar were searched from inception to 5 May 2022. Rat and mouse experiments assessing the therapeutic effects of β-asarone for AD were included. Primary outcomes were neuroethology, including escape latency and times of crossing platform. Second outcomes were cell apoptosis, including Bax and Bcl-2. The weighted mean difference (WMD) was generated for continuous variables. The relative outcomes were analyzed with the aid of Get Data Graph Digitizer 2.26 and software STATA version 16.0 MP.

**Results:** For the primary endpoint, compared with the modeling group, β-asarone significantly decreased the escape latency (WMD = -12.61, 95% CI: -18.66 to -6.57) and increased the times of crossing platform (WMD = 1.50, 95% CI: 0.31–2.70). For the secondary endpoint, β-asarone remarkably reduced the relative expression of the amyloid precursor protein (APP) (WMD = −2.25, 95% CI: −2.49 to −2.01), decreased the expression of the apoptosis-related protein, associated X protein (Bax) (WMD = −2.40, 95% CI: −3.51 to −1.29), lowered the expression of apoptosis-related protein, B-cell lymphoma-2 (Bcl-2) (WMD = 0.42, 95% CI: 0.38–0.46), and decreased the signal pathway-related proteins, phosphatidylinositol-3-kinase/protein kinase B (PI3K/AKT) (WMD = −0.70, 95% CI: −0.93 to −0.47) over the control group.

**Conclusion:** β-asarone spectacularly improved the learning ability and memory in rats and mice, which might be correlated with its potential neuroprotective effect through multiple signaling pathways.

## Introduction

Alzheimer’s disease (AD) is a degenerative disease of the central nervous system. Its pathological features mainly include extracellular amyloid beta (Aβ) deposition, intracellular neurofibrillary tangles (NFT), and neuronal loss. The typical clinical symptoms are irreversible cognitive decline and behavioral abnormalities.

There are currently more than 30 million cases of AD worldwide and this is expected to reach approximately 100 million cases by 2050 (González et al., 2019). It is estimated that 5.8 million Americans have AD, a number that is likely to triple by 2060 (Matthews et al., 2019). AD progression results in enormous morbidity and mortality and costs between $150 and $215 billion annually (Hurd et al., 2013). Indisputably, it is critical to investigate the pathogenesis of AD and find its optimal therapy.

At present, the clinical first-line drugs encompass donepezil hydrochloride tablets, memantine, rivastigmine etc., according to Diagnosis and treatment of Alzheimer’s disease (2022) (Author Anonymous, 2022). Meanwhile, in Chinese guidelines, traditional Chinese medicine (TCM) was recommended including AGS, Ginseng Radix, Angelica sinensis, Polygalae Radix, and Polygoni Multiflori Radix, indicating the validated effect on AD. Though the radical mechanisms of TCM in treating neurodegenerative diseases comprising AD, Parkinson’s disease (PD), multiple sclerosis (MS), Huntington’s disease (HD), amyotrophic lateral sclerosis (ALS), and Friedreich’s ataxia (FRDA), were not well elucidated, a fair number of studies have highlighted their potential in ameliorating oxidative stress, neuroinflammation, mitochondrial dysfunction, calcium disorder, autophagy, etc.

Therefore, as a widely acknowledged supplementary and alternative medicine, TCM should be recognized as efficient therapy that could go hand in hand with Western medicine in treating the aforementioned diseases. The typical paradigm is Tripterygium wilfordii in treating rheumatoid arthritis (RA), which is recommended in rife Western guidelines (Jiang, 2020).

**FIGURE 1:**
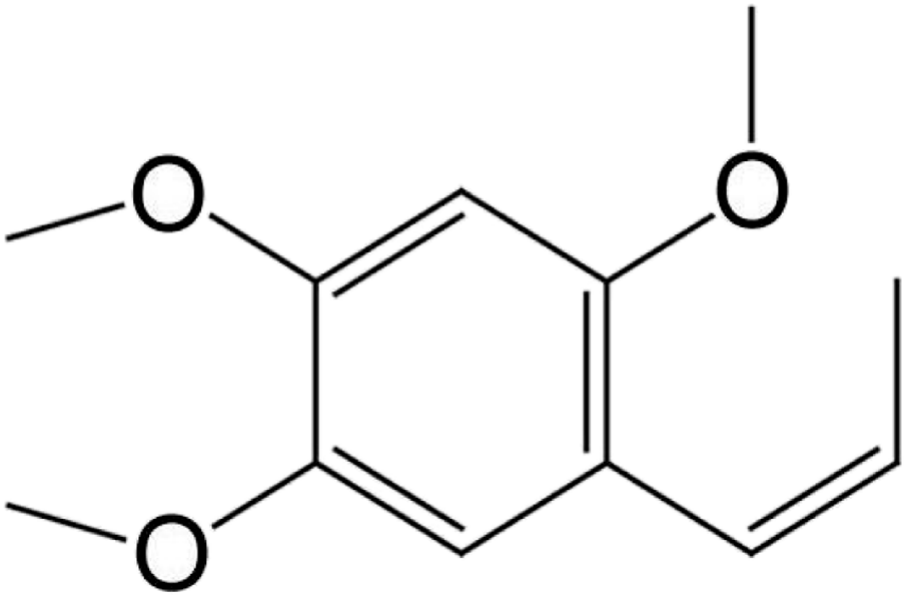
Chemical structure of β-asarone.

Hence, referring to RA, we conceive that β-asarone (Figure 1), which was purified from AGS, could be explored for its efficacy and potential mechanism in treating AD. It has been reported (Zhang, 2016) that β-asarone can act on multiple AD-related targets, including neuronal protection, promotion of BDNF synthesis and secretion, inhibition of Aβ aggregation, reduction of Aβ plaque formation, promotion of neuronal regeneration, and inhibition of inflammatory response. In addition, although β-asarone is small in polarity and can easily pass through the blood–brain barrier which has the characteristics of fast absorption and distribution, its oral bioavailability is low, the drug elimination rate in plasma is fast, and the half-life is short (Xu et al., 2020).

However, the efficacy and mechanism of β-asarone in the treatment of AD have not been systematically evaluated and summarized. Consequently, we decided to meticulously carry out this meta-analysis to coalesce data from individual studies to gather a larger sample size to investigate the efficacy of β-asarone in treating AD, and to separately explore the effect of β-asarone in certain pathways. Meanwhile, the preclinical data are summarized to lay the foundation for the development and application of new drugs. It is hoped that experts and scholars in this field pay more attention to the important role of TCM in disease treatment.

## Methods

The systematic review was conducted by the preferred reporting item (PRISMA) guidelines for systematic review and meta-analysis.

### Search strategy

The literature was retrieved from independent databases including PubMed, Embase, Scopus, Cochrane Library, BIOSIS Previews, Web of Science, EBSCO, and Google Scholar. The Mesh terms were (“β-asarone” OR “beta-asarone”) AND (“Alzheimer Dementia” OR “Alzheimer Dementias” OR “Alzheimer’s Disease” OR “Alzheimer Type Dementia” OR “Alzheimer-Type Dementia (ATD)" OR “Alzheimer Type Senile Dementia” OR “Primary Senile Degenerative Dementia” OR “Alzheimer’s Diseases”) AND (“Animal Models” OR “Laboratory Animal Models” OR “Animal Models, Laboratory” OR “Models, Laboratory Animal” OR “Experimental Animal Models” OR “Animal Models, Experimental” OR “Models, Experimental Animal").

### Inclusion and exclusion criteria

Studies in line with the following inclusion criteria were enrolled for this research:

1) Participants: experimental animals, including mice and rats.
2) Invention: containing only β-asarone.
3) Results: effect of β-asarone on animal models of AD, including neurobehavior.

Studies that met the following criteria were also excluded:

1) Combined use of other drugs;
2) Non-animal studies;
3) Case reports, reviews, clinical experience or trial, and review articles;
4) Similar and repeat studies;
5) Single arm studies.

### Data extraction and quality assessment

Two investigators (ZT and YJ) independently searched, assessed, and extracted data from the literature. Any discrepancy was arbitrated by a senior investigator (DXY).

Data were collected including first author, publication year, animal type, months of age, gender, body weight, intervention, and outcome indicators.

Primary outcomes were indices related to neuroethology, including escape latency, time of crossing platform, etc.

Secondary outcomes were indices related to cell apoptosis, including Bax and Bcl-2. The articles’ quality was evaluated using SYRCLE’s risk of bias tool for randomized controlled studies (RCTs).

If the results are tested at different times, the peak time point is utilized. Data on the highest dose of β-asarone across studies were extracted in the study. If the primary data are missing or presented graphically, we try to contact the author to obtain the original data. When no reply was received from the author, the values in the figure were measured using the digital ruler software.

### Statistical methods

The software utilized was STATA version 16.0 MP. The weighted mean difference (WMD) was generated as effect-size for continuous variants. Odds ratios (ORs) were generated for dichotomous variants. If only figures were presented, two researchers independently used Get Data Graph Digitizer ver. 2.26 to extract data and compute the means^(25)^. If *I* ^*2*^ ≤ 50% and *p* > 0.01, a fixed effect model would be implemented, otherwise, a random effect model would be performed. If *I* ^*2*^ ≥ 75%, sensitive analysis, subgroup analysis, or the Galbraith plot would be carried out to eliminate heterogeneity. Publication bias was assessed by Egger’s test. Probability value *p* < 0.05 was considered statistically significant.

## Results

### Study selection

**FIGURE 2:**
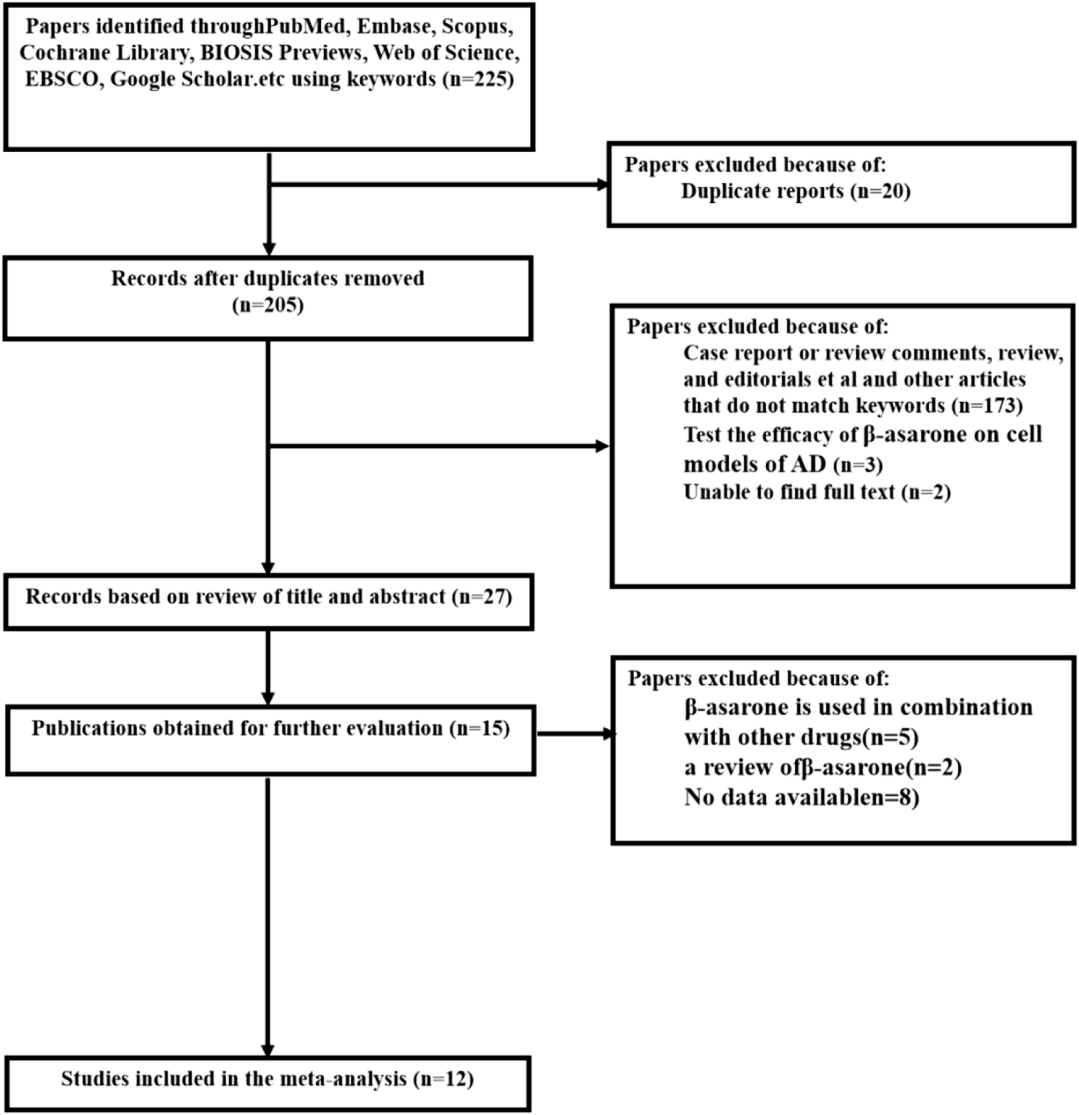
Literature search diagram.

After an initial search of eight databases, a total of 225 potential publications were identified. After removing duplicated and irrelevant articles, 205 records were retained. By screening the titles and abstracts, 178 studies were excluded because they were conferences, clinical trials, or review articles. After reading the remaining full-text articles, 15 studies were excluded, for at least one of the following reasons (González et al., 2019): no data were available (Matthews et al., 2019); not a rat or mouse model (Hurd et al., 2013); and combined with other drugs. Ultimately, we selected 12 eligible studies. A flowchart of the study selection process is shown in Figure 2 (Geng et al., 2010; Liu et al., 2010; Li et al., 2012; Lin, 2012; Tian, 2012; Yu, 2013; Li, 2015; Deng et al., 2016; Guo et al., 2017; Ma et al., 2017; Wang et al., 2017; Deng et al., 2020).

### Characteristics of included studies

Rats (*n* = 6) and mice (*n* = 6) were divided into four strains: SD rats (*n* = 5), Wistar rats (*n* = 1), SAMP8 mice (*n* = 1), and APPswe/ps1de9 double transgenic mice (*n* = 5). Among the studies, six articles used male animals, one used female animals, five used males and females, and the remaining nine did not mention the gender of the animals used. The number and weight range of animals used in these studies varied. The weight of rats ranged from 180–300 g (Table 1).

**TABLE 1:** Characteristics of the 12 included animal studies.

Author (years)	Species and modeling approach	Months of age/body weight	β-asarone administration (Ad: administration route)	Endpoint	Pharmacological activity (mechanisms)
Ma et al., 2017	Male	Adult	Dosage: 12.5, 25, 50mg/(kg·d)	①	Improve cognitive impairment (GAP-43↓, PSD-95↓, SYP↑)
	SD rats	260–280 g	Ad: i.g.; duration: 4 weeks		
	Aβ_1-42_				
Guo, et al., 2017	Half male and half female	3 months	Dosage: 15, 10, 5 mg/(kg·d)	③	Alleviated learning and memory deficits (H2A1-H↑, H2B2-E↑, H2B1-F/J/L↑)
	APP/PS1 swe/dE9 double transgenic mice	-	Ad: i.g.; duration: 12 weeks		
Li, et al., 2015	Half male and half female	5 months	Dosage: 21.2 mg/(kg·d)	①	Protect neurons
	APP/PS1 swe/dE9 double transgenic mice	-	Ad: i.g.; duration: 8 weeks		
Yu, et al., 2013	Half male and half female	4–5 months	Dosage:21.2,42.4,84.8 mg/(kg·d)	①②④⑤⑥	Improves memory and cognitive learning
	APP/PS1 swe/dE9 double transgenic mice	-	Ad: i.g.; duration: 10 weeks		
Tian, et al., 2012	Male	-	Dosage: 5, 15, 45 mg/(kg·d)	①②	Perfect learning and memory ability; neuronal protection (Smad2↑, Bax↓)
	SD rats	200–250 g	Ad: i.g.; duration: 4 weeks		
	IBO				
Lin, et al., 2012	Male	6 months	Dosage: 42.4, 84.8 mg/(kg·d)	①②⑤	Improve learning disabilities (Aβ42↓, Bax↓, tau levels↓, Bcl-2↑)
	SAMP8 mice	25 ± 5 g	Ad: i.g.; duration:8 weeks		
Deng, et al., 2020	Half male and half female	3 months	Dosage:10, 20, 40 mg/(kg·d)	③	Scavenging Aβ accumulation and inhibiting autophagy activity
	APP/PS1 swe/dE9 double transgenic mice	20–25 g	Ad:i.g.; Duration:4 weeks		
Wang, et al., 2017	Male	Adult	Dosage: 10, 20, 30 mg/(kg·d)	①②	Alleviate the pathological state of AD (HIF-1α levels↓, oxidative stress↓, (SOD↑, MDA↓)
	SD rats	280 ± 20 g	Ad: i.g.; duration:4 weeks		
	Aβ_1-42_				
Deng, et al., 2016	Half male and half female	3 months	Dosage: 10 mg/(kg·d)	⑥	Some behavioral disorders were alleviated (AChE↓, Aβ42↓, APP↓, Beclin-1↓)
	APP/PS1 swe/dE9 double transgenic mice	20–25 g	Ad: i.g.; duration: 4 weeks		
Li, et al., 2012	Female	-	Dosage: 25, 50, 100 mg/(kg·d)	①	Enhance the learning and memory ability of AD rats (ET-1 mRNA↓)
	Wistar rats	150–180 g	Ad: i.g.; Duration:2 weeks		
	AlCl_3_ and D-gal				
Liu, et al., 2010	Male	-	Dosage: 12.5, 25, 50 mg/(kg·d)	④	Anti-apoptosis (p-c-Jun↓, Bad↓, Bax↓, caspase-9↓)
	SD rats	220–240 g	Ad: i.g.; duration: 4 weeks		
	Aβ_1-42_				
Geng, et al., 2010	Male	-	Dosage: 12.5, 25, 50 mg/(kg·d)	②⑤	Improved spatial memory (JNK↓, Bcl-w↑, Bcl-2↑, caspase-3↓)
	SD rats	220–240 g	Ad: i.g.; duration:4weeks		
	Aβ_1-42_				

①escape latency; ②times of crossing platform; ③relative expression of APP; ④expression of apoptosis-related protein Bax; ⑤expression of apoptosis-related protein Bcl-2; ⑥signal pathway-related proteins PI3K/AKT.

### Study quality

SYRCLE shows that the overall quality of the articles was medium and high, and a small part had publication bias (Table 2).

**TABLE 2:** Results of SYRCLE’s risk of bias.

Study (year)	Sequence to produce	Baseline characteristics	Distribution of hidden	Randomize animal placement	Randomize animal placement	Evaluation of random outcomes	Result evaluator blind	Incomplete data reporting	Selective result report	Selective result report
Ma, et al., 2017	NC	Y	N	Y	NC	NC	NC	NC	Y	Y
Guo, et al., 2017	NC	Y	N	Y	NC	NC	NC	NC	Y	Y
Li, et al., 2015	NC	N	N	Y	NC	NC	NC	Y	Y	Y
Yu, et al., 2013	Y	N	N	Y	NC	NC	NC	NC	Y	Y
Tian, et al., 2012	Y	N	N	Y	NC	NC	NC	NC	Y	Y
Lin, et al., 2012	N	N	N	Y	NC	NC	NC	NC	Y	Y
Deng, et al., 2020	NC	Y	N	Y	NC	NC	NC	NC	Y	Y
Wang, et al., 2017	Y	Y	N	Y	NC	NC	NC	NC	Y	Y
Deng, et al., 2016	NC	Y	N	Y	NC	NC	NC	NC	Y	Y
Li, et al., 2012	NC	N	N	Y	NC	NC	NC	NC	Y	Y
Liu, et al., 2010	NC	Y	N	Y	NC	NC	NC	NC	Y	Y
Geng, et al., 2010	NC	Y	N	Y	NC	NC	NC	NC	Y	Y

Y=yes; N=no; NC, not clear.

### Effectiveness

#### Behavioral test analysis (primary endpoint)

**FIGURE 3:**
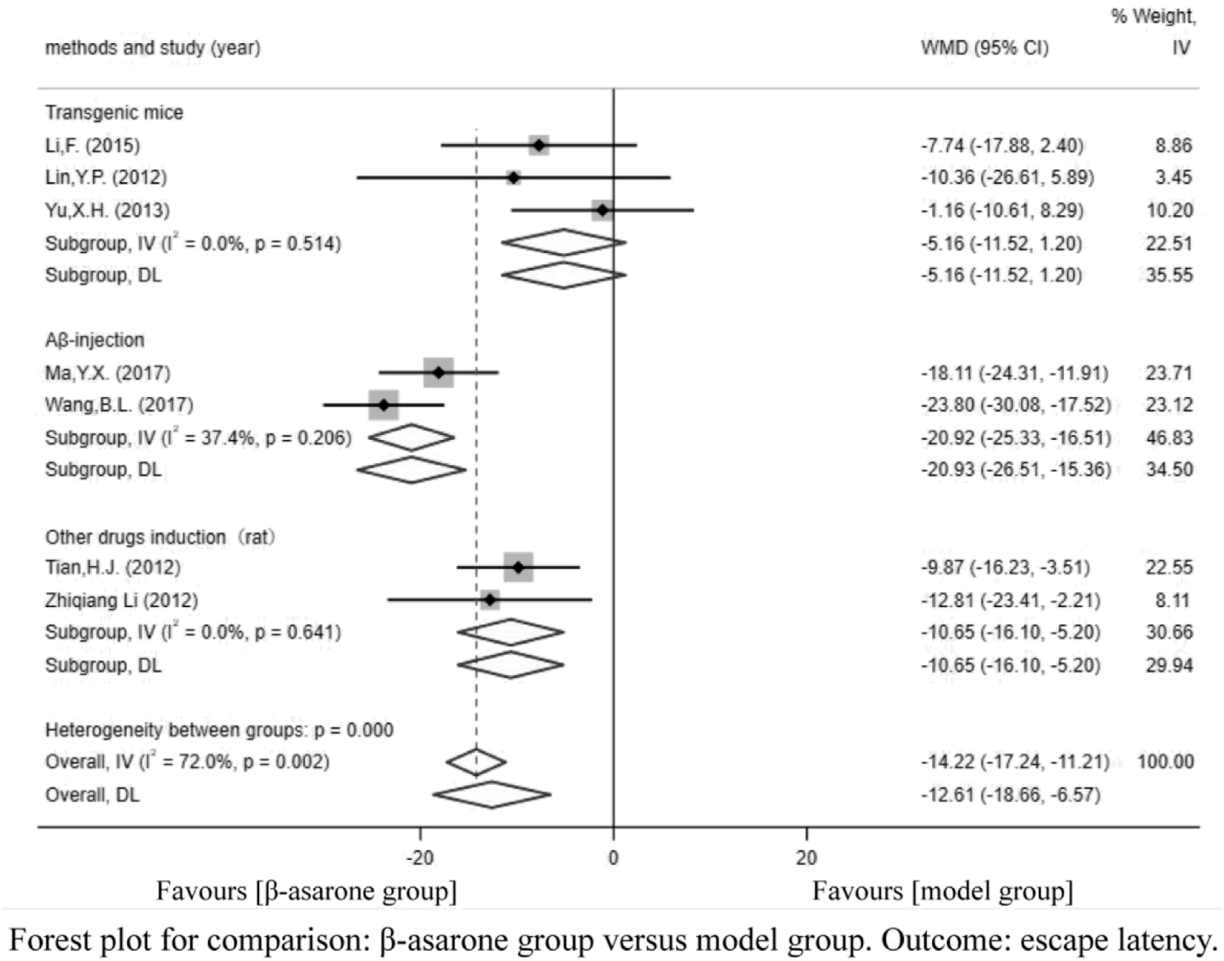
Forest plot for comparison: β-asarone group versus model group. Outcome: escape latency.

Of the 12 articles, seven (Li et al., 2012; Lin, 2012; Tian, 2012; Yu, 2013; Li, 2015; Ma et al., 2017; Wang et al., 2017) reported escape latency. Compared with modeling groups, β-asarone groups showed a significant decline in escape latency by 12.61 s (95% CI: -18.66 to -6.57). According to the modeling approaches, the studies were classified into three subgroups, namely, Aβ-injection, transgenic mice, and other drugs induction (rat). The results of the subgroup were 20.93 s (95% CI: −26.51 to −15.36), -5.16 s (95% CI: −11.52 to 1.20), and −10.65 s (95% CI: −16.10 to −5.20) in the β-asarone groups over the modeling groups (Figure 3).

**FIGURE 4:**
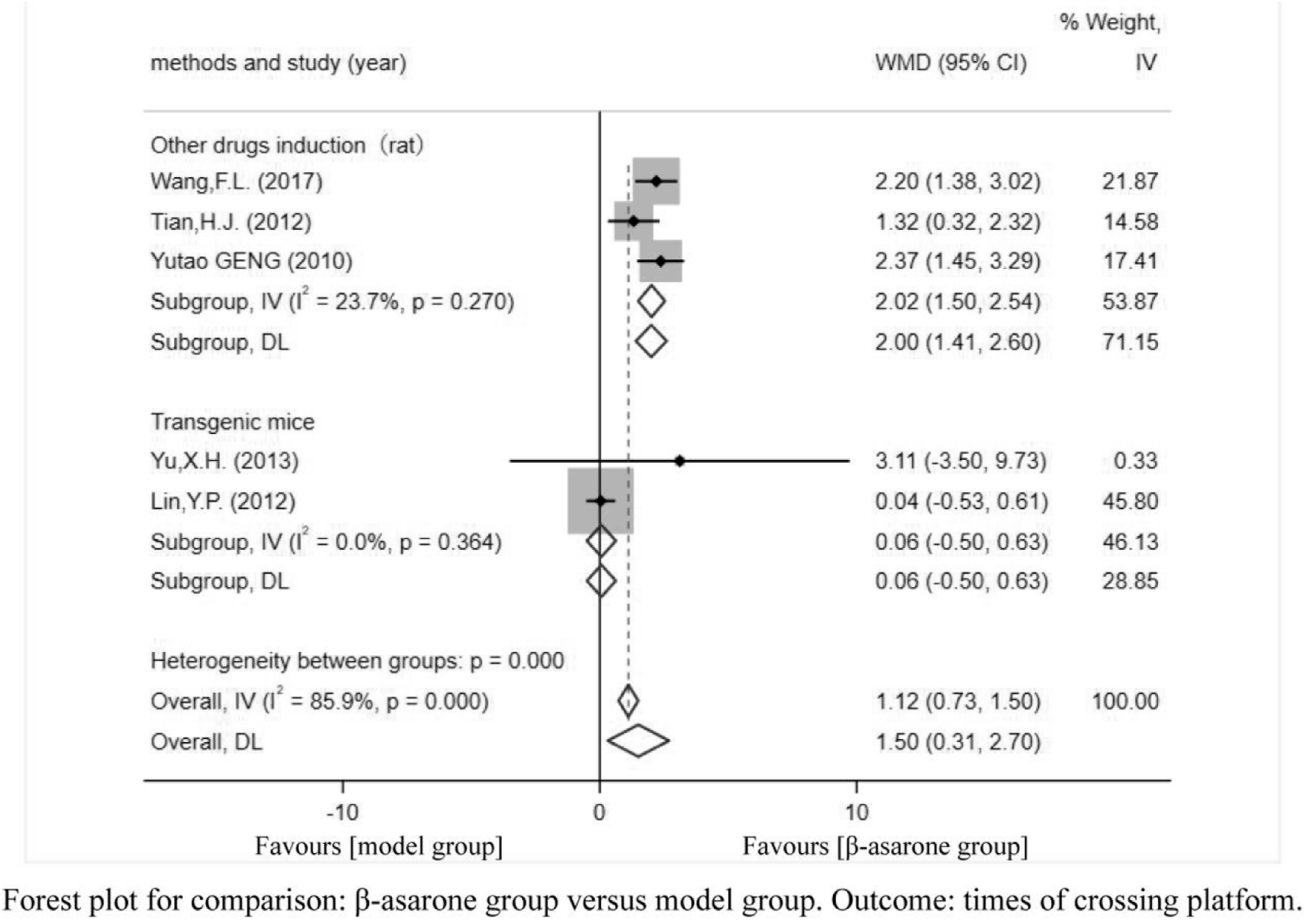
Forest plot for comparison: β-asarone group versus model group. Outcome: times of crossing platforms.

Of the 12 articles, five (Geng et al., 2010; Lin, 2012; Tian, 2012; Yu, 2013; Wang et al., 2017) reported the times of crossing platforms. Compared with modeling groups, β-asarone groups showed a significant increment in the times of crossing platforms by 1.50 s (95% CI: 0.31–2.70). According to the modeling approaches, the studies were classified into two subgroups, namely, transgenic mice and other drugs induction (rat). The results of the subgroup were 0.06 times (95% CI: -0.50–0.63) and 2.00 times (95% CI: 1.41–2.60) (Figure 4).

#### Neuroprotective mechanism (secondary endpoint)

##### The inhibition of abnormal protein aggregation

**FIGURE 5:**
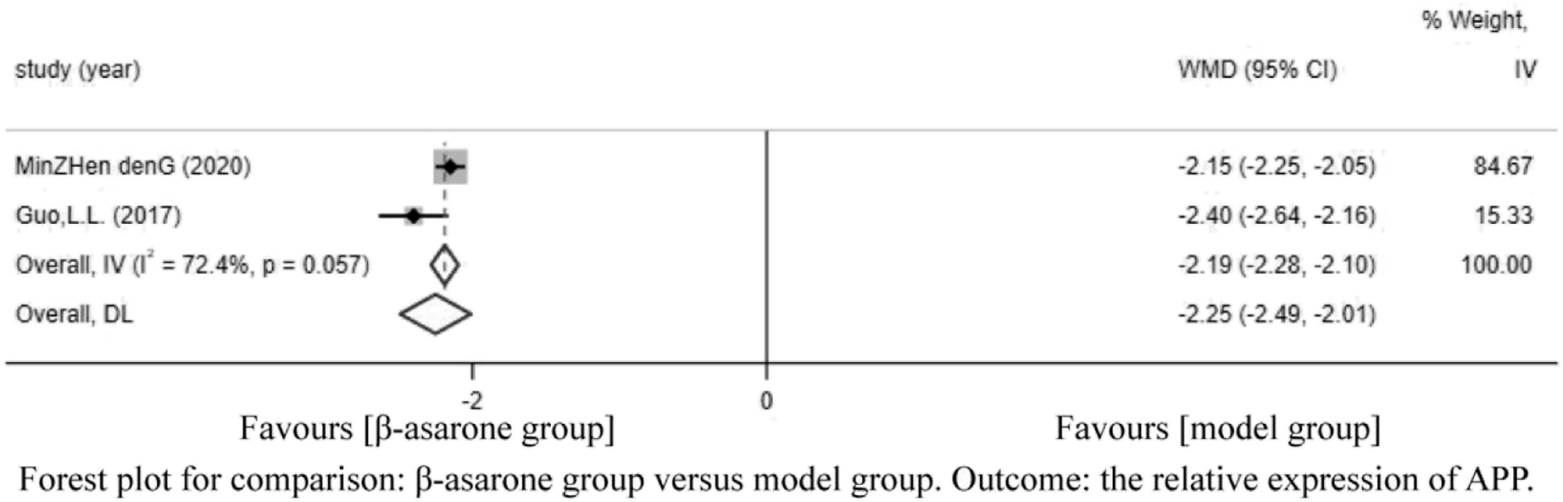
Forest plot for comparison: β-asarone group versus model group. Outcome: the relative expression of APP.

Of the 12 articles, two (Guo et al., 2017; Deng et al., 2020) reported the relative expression of APP. Compared with modeling groups, β-asarone groups showed a significant decrease in the relative expression of APP (WMD = −2.25, 95% CI: −2.49 to −2.01) (Figure 5).

##### The inhibition of neuronal apoptosis

**FIGURE 6:**
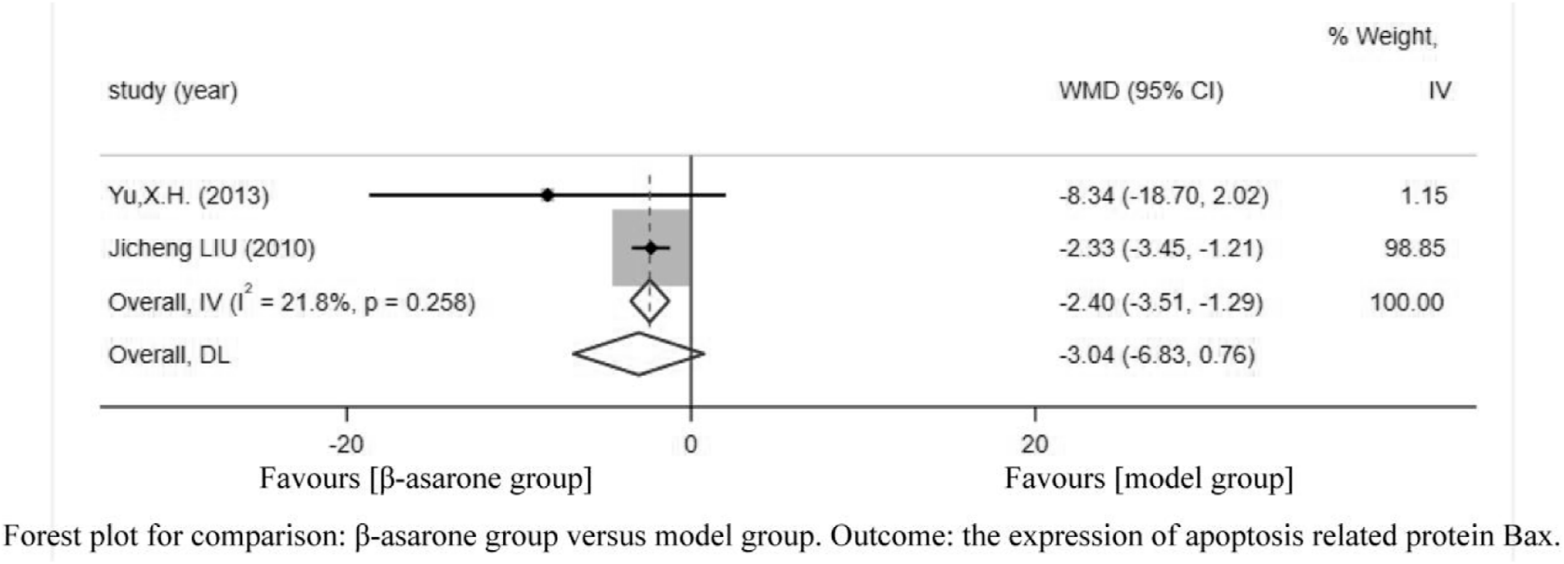
Forest plot for comparison: β-asarone group versus model group. Outcome: the expression of apoptosis-related protein Bax.

Of the 12 articles, two (Liu et al., 2010; Yu, 2013) reported the expression of apoptosis-related protein Bax. Compared with modeling groups, β-asarone groups showed a significant decrease in the expression of apoptosis-related protein Bax (WMD = −2.40, 95% CI: −3.51 to −1.29) (Figure 6).

**FIGURE 7:**
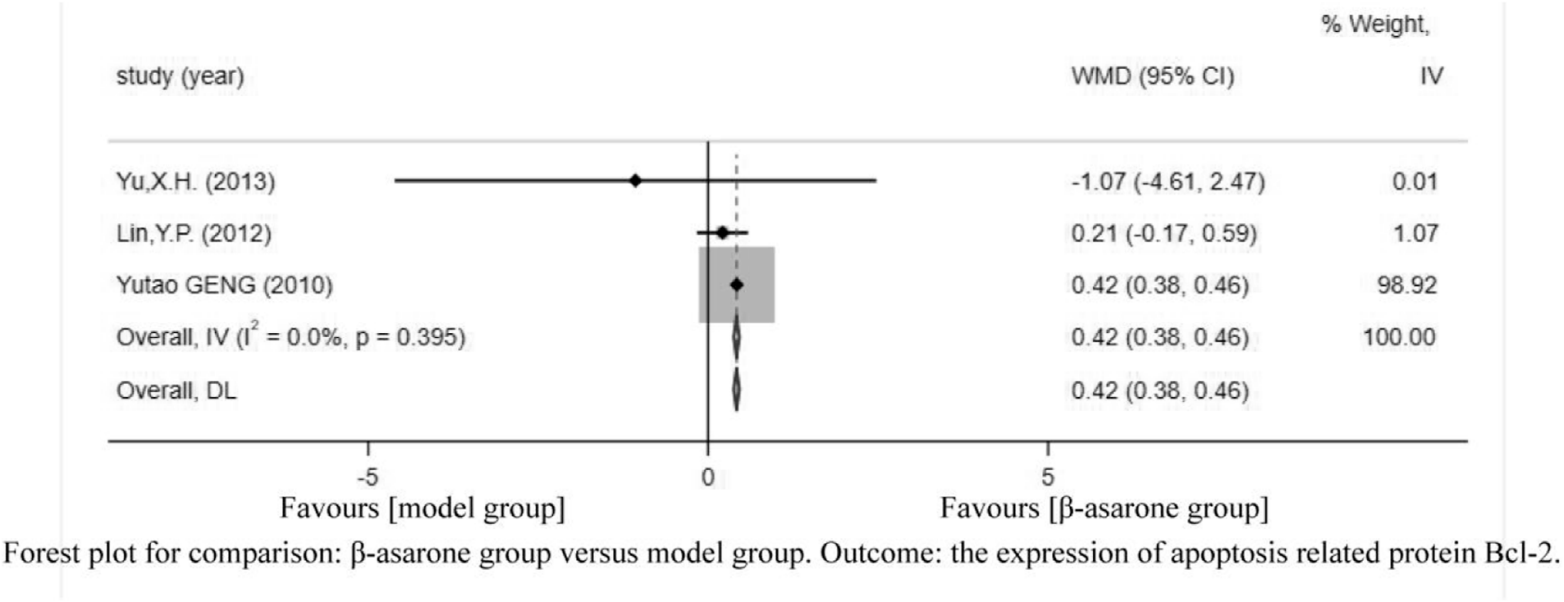
Forest plot for comparison: β-asarone group versus model group. Outcome: the expression of apoptosis-related protein Bcl-2.

Of the 12 articles, three (Geng et al., 2010; Lin, 2012; Yu, 2013) reported the expression of apoptosis-related protein Bcl-2. Compared with modeling groups, β-asarone groups showed a significant increase in the expression of apoptosis-related protein Bcl-2 (WMD = 0.42, 95% CI: 0.38–0.46) (Figure 7).

**FIGURE 8:**
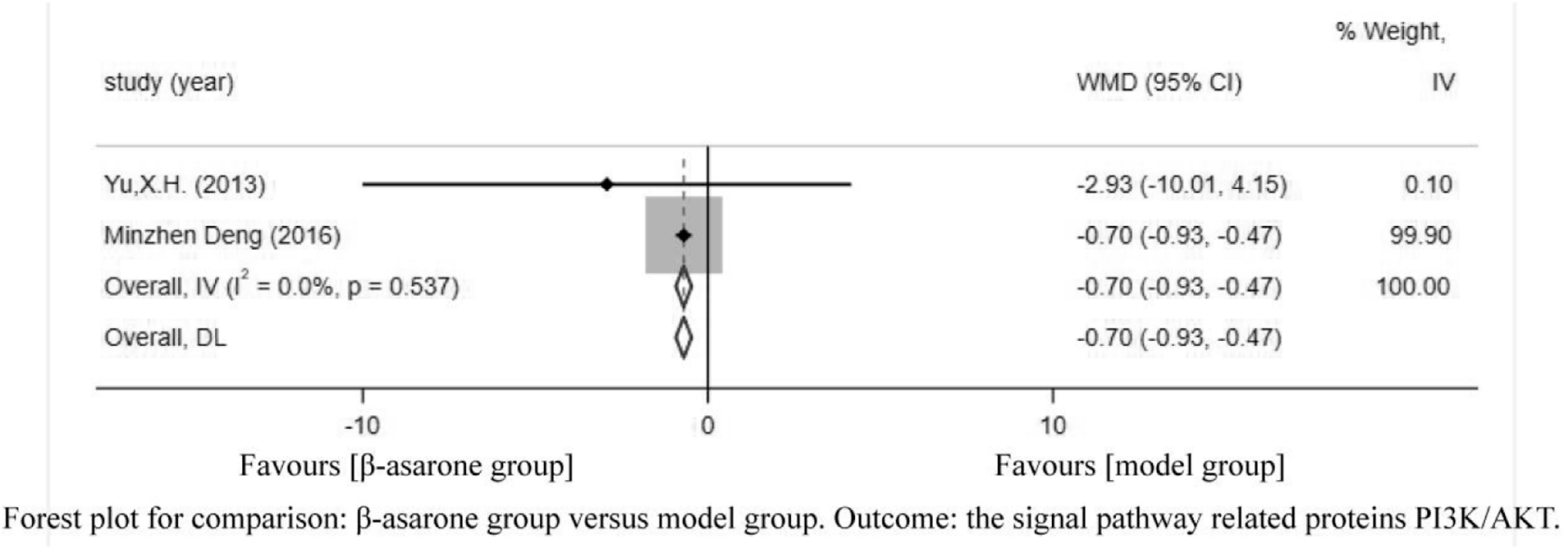
Forest plot for comparison: β-asarone group versus model group. Outcome: the signal pathway-related proteins PI3K/AKT.

Of the 12 articles, two (Yu, 2013; Deng et al., 2016) reported the signal pathway-related proteins PI3K/AKT. Compared with modeling groups, β-asarone groups showed a significant increase in the signal pathway-related proteins PI3K/AKT (WMD = -0.70, 95% CI: -0.93 to -0.47) (Figure 8).

### The suppression of oxidative stress

One study (Wang et al., 2017) showed that β-asarone increased the expression of SOD and decreased the expression of HIF-α, WMDA, and ROS in experimental animal models of AD.

### The inhibition of autophagy

One study (Deng et al., 2020) demonstrated that β-asarone decreased Beclin-1 and LC3A/B levels and the number of senile plaques, and enhanced the expression of P_62_ in experimental animal models of AD.

### The restoration of mitochondrial dysfunction

One study (Han et al., 2020) manifested that β-asarone reduced Aβ_1–42_ levels and ameliorated cognitive deficits by inhibiting PINK1-Parkin-mediated mitophagy.

### The restoration of synaptic functional plasticity in neurons

One study (Liu et al., 2016) demonstrated that β-asarone significantly increased the expression of key proteins of synaptic plasticity (SYN, PSD95, GluR1, etc.). In addition, β-asarone could restore synaptic plasticity by controlling calcium overload.

### Source analysis of heterogeneity

**FIGURE 9:**
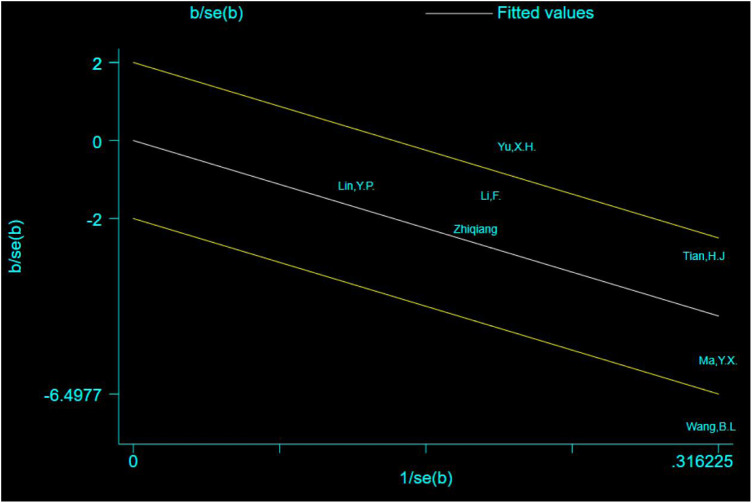
Galbraith plot for the outcome of escape latency.

The Galbraith plot was used to conduct qualitative analysis for the heterogeneity of the outcome of escape latency and the results showed that except for two studies that fell squarely on the regression line at the upper limit of the 95% confidence interval of the combined effect size, all the other studies fell within the regression line range of the upper and lower limits. This suggests that the effect sizes of the independent studies are likely to be homogeneous (Figure 9).

**FIGURE 10:**
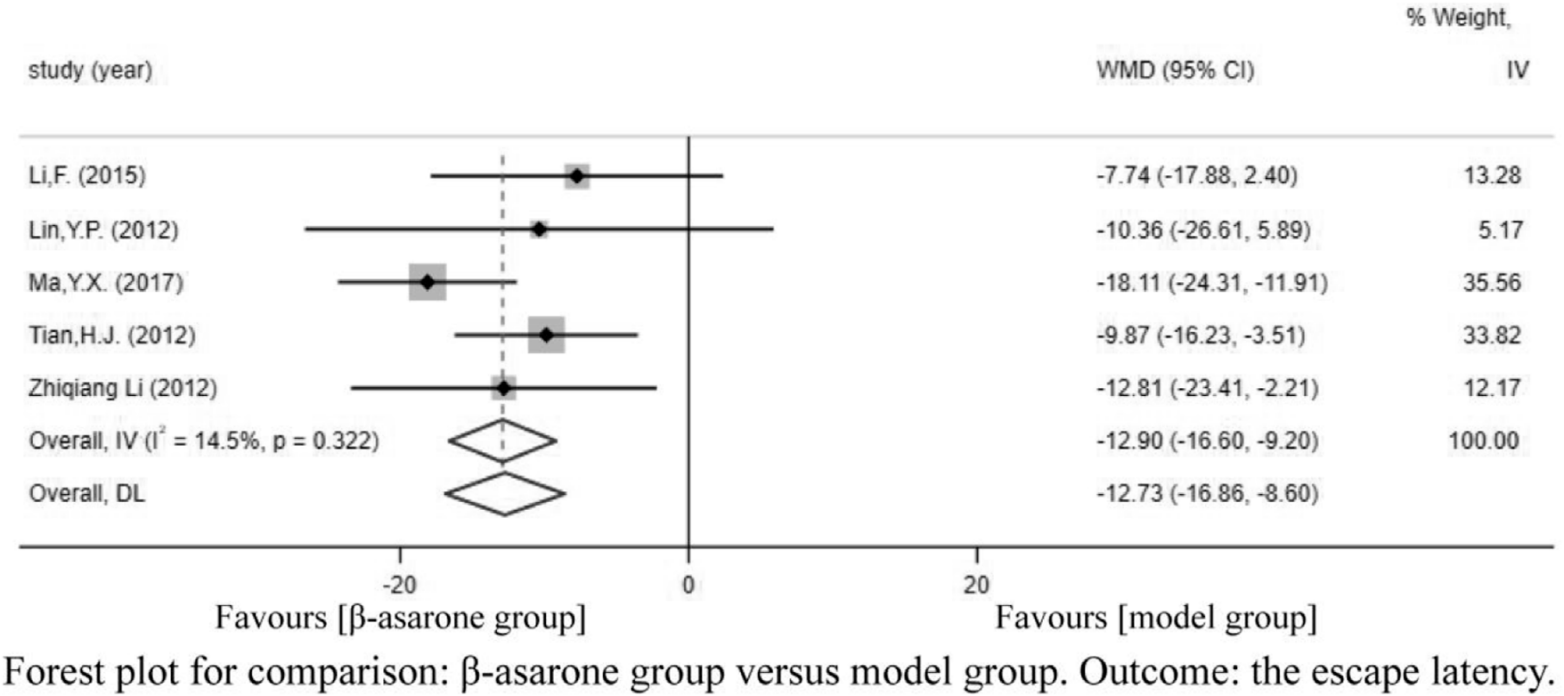
Forest plot for comparison: β-asarone group versus model group. Outcome: escape latency.

Excepting for two studies (Yu, 2013; Wang et al., 2017) that fell squarely on the regression line at the upper limit of the 95% confidence interval of the combined effect size, repainting of the forest for all the other studies showed that heterogeneity was significantly eliminated and only homogeneity existed (Figure 10) **(** Li et al., 2012).

**FIGURE 11:**
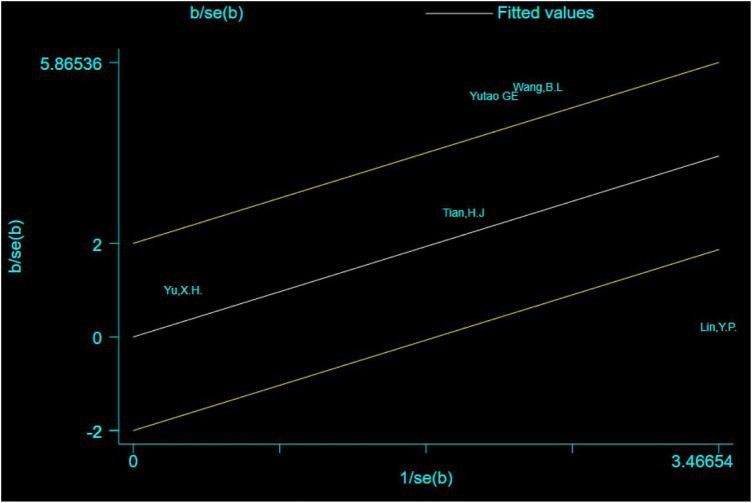
Galbraith plot for the outcome of the times of crossing platforms.

The Galbraith plot was used to conduct qualitative analysis for the heterogeneity of the outcome of the times of crossing platforms, and the results showed that except for three studies that fell squarely on the regression line at the upper limit of the 95% confidence interval of the combined effect size, all the other studies fell within the regression line range of the upper and lower limits. This suggests that the effect sizes of the independent studies are likely to be homogeneous (Figure 11).

**FIGURE 12:**
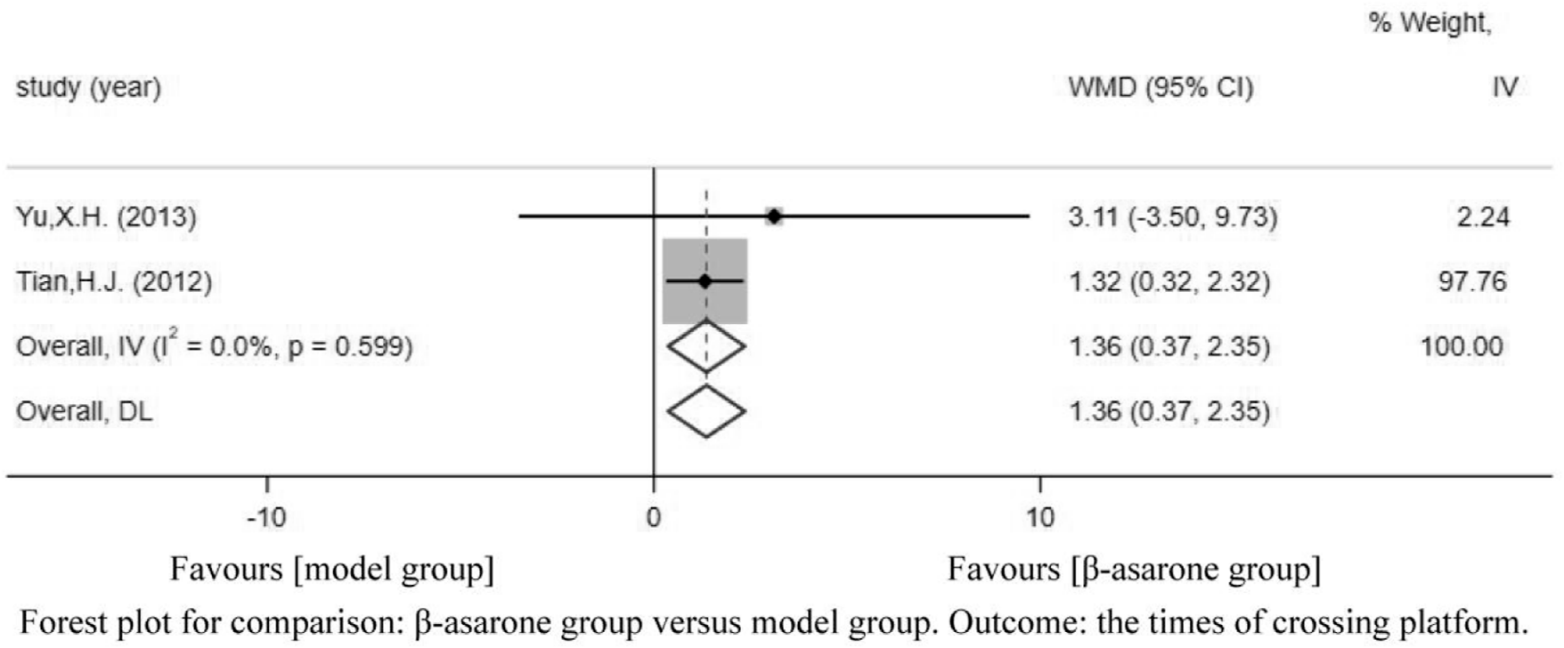
Forest plot for comparison: β-asarone group versus model group. Outcome: the times of crossing platforms.

Except for three studies (Geng et al., 2010; Lin, 2012; Wang et al., 2017) that fell squarely on the regression line at the upper limit of the 95% confidence interval of the combined effect size, repainting of the forest for all the other studies showed that heterogeneity was significantly eliminated and only homogeneity existed (Figure 12).

**FIGURE 13:**
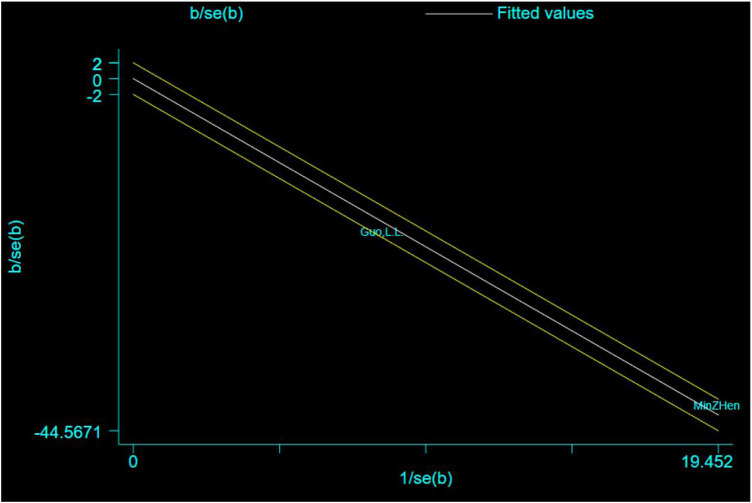
Galbraith plot for the outcome of the relative expression of amyloid precursor protein (APP).

The Galbraith plot was used to conduct qualitative analysis for the heterogeneity of the relative expression of the amyloid precursor protein (APP), and the results showed that all studies fell within the regression line range of the upper and lower limits (Figure 13).

### Publication bias

Publication bias was examined by Egger’s regression (*p* > 0.05) for two outcomes about escape latency and the expression of apoptosis-related protein Bcl-2. The result showed that the *p-*value of bias was 0.513 and 0.120. It hinted that there was no obvious publication bias. In addition, all indicators were tested for publication bias as required, but only two indicators could get results, and the remaining indicators could not be tested due to the small number of literature.

## Discussion

### Summary of results

To the best of our knowledge, this is the first meta-analysis of a preclinical study to determine the effects of β-asarone for experimental AD in rodent models owing to the dearth of RCT. We enhanced the findings that β-asarone had neuroprotective effects in experimental AD models. Additionally, other studies on the efficacy of β-asarone in rodent models have addressed similar results to ours (Geng et al., 2010; Lin, 2012; Tian, 2012; Yu, 2013; Li, 2015).

### Correlation of clinical results with pathology

Previous literature has reported that β-asarone could be targeted to some pathways including JAK/STAT, MAPK, and NF-κB which were associated with AD. However, our study uncovered more pathways incorporating PI3K/AKT, Wnt/β-catenin, PINK1/Parkin, Notch, etc. relevant to AD, resulting in improving behavior performance, inhibiting abnormal accumulation of amyloid protein, suppressing neuronal apoptosis, attenuating oxidative stress, restraining autophagy, restoring mitochondrial dysfunction, promoting brain-derived neurotrophic factor (BDNF) synthetic secretion, inhibiting neuroinflammation, and enhancing synaptic functional plasticity of neurons. Macroscopically, the aforementioned mechanisms contribute to the amelioration of cognitive, learning, and memory functions. Hence, we augmented the correlation of clinical results with pathology.

### Implications for clinical settings

Memantine is a receptor antagonist of N-methyl-D-aspartate (NMDA), and its pathways are different from that of β-asarone. It plays a role in the treatment of advanced AD by regulating the activity of glutamic acid. Theoretically, adjuvant therapy of memantine and β-asarone is reasonable.

Pragmatically, there indeed existed a study combining memantine and β-asarone; the result showed that this couple improved the efficacy of memantine in the treatment of moderate-to-severe AD (Chang and Teng, 2018). To be more precise, after 12 weeks of treatment, the average MMSE score, ADL score, and CDR score of the two groups (memantine group and memantine+β-asarone group) of patients improved significantly. However, the average MMSE score of the memantine+β-asarone group was significantly higher than that of the memantine group, the average ADL score was prominently lower than that of the control group, and the average CDR score was considerably lower than that of the control group. At the same time, the incidence of adverse events was similar in both groups. Subgroup analysis showed that the memantine+β-asarone group was inclined to benefit moderate AD patients in men aged 60–74 years.

The efficacy of β-asarone orchestrated with other Western agents, such as donepezil hydrochloride tablets, memantine, and rivastigmine, entail more studies to validate.

In terms of cost-effectiveness, taking memantine as an example, the median daily drug price of it for AD is less than $2.76 in China (https://www.fda.gov/). Since β-asarone is not expensive in China, the total price might be affordable and the benefit of the combination of the two agents should outweigh their prices.

Altogether, given the current limited evidence, we recommend memantine +β-asarone as the most promising clinical option for AD treatment considering clinical feasibility, cost-effectiveness, and safety. However, the drug–drug interactions between Western agents and β-asarone and adverse events of those regimens may necessitate clinical trials with larger sample sizes to confirm.

### Adverse reaction analysis

Toxicological studies have shown that β-asarone can cause hepatocellular carcinoma and may have mutagenic, genotoxic, and teratogenic effects in animal models. However, clinical studies did not observe the aforementioned toxicity, indicating that not all results from animal studies could be extended to humans.

Moreover, in terms of approval of any drug, strict phase I, II, and III RCTs of β-asarone should be carried out to fully ensure efficacy and safety.

As we all know, before the application of new traditional Chinese medicine (TCM) in clinical practice, it is necessary to explore the effect and mechanism of drug treatment of diseases, then conduct cell experiments *in vitro*, conduct *in vivo* experiments with animals to determine the efficacy and safety, conduct pharmacokinetic experiments, and finally apply to clinical trials. In recent years, TCM has been rapidly developing purified compounds with modern technology. The era of taking herbs and stewing them in a stew has passed. As the active ingredients in TCM become more clearer, it is natural that further studies on the pharmacokinetics of traditional Chinese medicine will occur. We should view TCM from the perspective of development.

### Limitations

#### This meta-analysis has several limitations.

First, current therapeutic models for AD are limited to rats and mice; such animals differ greatly from humans *in vivo* under the pathological microenvironment, which brings limited reference value to clinical treatment. Therefore, animal models which are similar to humans such as monkeys and apes can be considered in the future to better simulate the pathological conditions of clinical AD; second, this study is a meta-analysis, and the evidence chain cannot be formed, so only a scattered mechanism can be obtained; third, it is true that the amount of literature we have reviewed is relatively small, but this is all we can find, so we need more animal experiments to provide proof in the future.

## Conclusion

β-asarone spectacularly improved the learning ability and memory in rats and mice, which might be correlated with its potential neuroprotective effect through multiple signaling pathways. It is worth noting that there are many different drugs to improve Alzheimer’s disease (AD) from different pathways. As a supplement and alternative medicine, traditional Chinese medicine (TCM) targets multiple pathways which may be different from classical Western medicine, which may be orchestrated with Western medicine to materialize multiplying efficacy in AD patients.

## Data Availability

The original contributions presented in the study are included in the article/Supplementary Material; further inquiries can be directed to the corresponding author.
